# Data mining analyses for precision medicine in acromegaly: a proof of concept

**DOI:** 10.1038/s41598-022-12955-2

**Published:** 2022-05-28

**Authors:** Joan Gil, Montserrat Marques-Pamies, Miguel Sampedro, Susan M. Webb, Guillermo Serra, Isabel Salinas, Alberto Blanco, Elena Valassi, Cristina Carrato, Antonio Picó, Araceli García-Martínez, Luciana Martel-Duguech, Teresa Sardon, Andreu Simó-Servat, Betina Biagetti, Carles Villabona, Rosa Cámara, Carmen Fajardo-Montañana, Cristina Álvarez-Escolá, Cristina Lamas, Clara V. Alvarez, Ignacio Bernabéu, Mónica Marazuela, Mireia Jordà, Manel Puig-Domingo

**Affiliations:** 1 Department of Endocrinology and Nutrition, Germans Trias I Pujol Research Institute (IGTP), Camí de les Escoles, s/n, 08916 Badalona, Catalonia Spain; 2 Department of Endocrinology/Medicine, CIBERER U747, ISCIII, Research Center for Pituitary Diseases, Hospital Sant Pau, IIB-SPau, Universitat Autònoma de Barcelona, Barcelona, Spain; 3 Biomedical Research Networking Center in Rare Diseases (CIBERER), Institute of Health Carlos III (ISCIII), Madrid, Spain; 4 Department of Endocrinology and Nutrition, Germans Trias I Pujol University Hospital, Badalona, Spain; 5 Department of Endocrinology, Hospital de La Princesa, Universidad Autónoma de Madrid, Instituto Princesa, Madrid, Spain; 6 Department of Endocrinology, Son Espases University Hospital, Palma de Mallorca, Balearic Islands Spain; 7 Department of Neurosurgery, Germans Trias I Pujol University Hospital, Badalona, Spain; 8 Department of Pathology, Germans Trias I Pujol University Hospital, Badalona, Spain; 9 Hospital General Universitario de Alicante-Institute for Health and Biomedical Research (ISABIAL), Alicante, Spain; 10 Department of Clinical Medicine, Miguel Hernández University, Elche, Spain; 11 Anaxomics Biotech S.L., Barcelona, Spain; 12 Department of Endocrinology, Hospital Universitari Mutua Terrassa, Terrassa, Spain; 13 Department of Endocrinology, Hospital General Universitari Vall d’Hebron, Universitat Autònoma de Barcelona, Barcelona, Spain; 14 Department of Endocrinology, Hospital Universitari de Bellvitge, L’Hospitalet de Llobregat, Spain; 15 Endocrinology Department, Hospital Universitario Y Politécnico La Fe, Valencia, Spain; 16 Endocrinology Department, Hospital Universitario de La Ribera, Alzira, Spain; 17 Endocrinology Department, Hospital Universitario La Paz, Madrid, Spain; 18 Endocrinology Department, Hospital General Universitario de Albacete, Albacete, Spain; 19 Neoplasia & Endocrine Differentiation P0L5, Centro de Investigacion en Medicina Molecular Y Enfermedades Cronicas (CIMUS), Instituto de Investigacion Sanitaria de Santiago (IDIS), Universidad de Santiago de Compostela (USC), Santiago, Spain; 20 Endocrinology Division, Complejo Hospitalario Universitario de Santiago de Compostela (CHUS)-SERGAS, Santiago de Compostela, Spain; 21 Department of Medicine, Autonomous University of Barcelona (UAB), Bellaterra, Spain

**Keywords:** Endocrinology, Endocrine system and metabolic diseases, Molecular medicine, Predictive markers

## Abstract

Predicting which acromegaly patients could benefit from somatostatin receptor ligands (SRL) is a must for personalized medicine. Although many biomarkers linked to SRL response have been identified, there is no consensus criterion on how to assign this pharmacologic treatment according to biomarker levels. Our aim is to provide better predictive tools for an accurate acromegaly patient stratification regarding the ability to respond to SRL. We took advantage of a multicenter study of 71 acromegaly patients and we used advanced mathematical modelling to predict SRL response combining molecular and clinical information. Different models of patient stratification were obtained, with a much higher accuracy when the studied cohort is fragmented according to relevant clinical characteristics. Considering all the models, a patient stratification based on the extrasellar growth of the tumor, sex, age and the expression of E-cadherin, *GHRL*, *IN1-GHRL*, *DRD2*, *SSTR5* and *PEBP1* is proposed, with accuracies that stand between 71 to 95%. In conclusion, the use of data mining could be very useful for implementation of personalized medicine in acromegaly through an interdisciplinary work between computer science, mathematics, biology and medicine. This new methodology opens a door to more precise and personalized medicine for acromegaly patients.

## Introduction

Acromegaly is typically diagnosed late, when the symptomatology is strikingly present^1,2^. Neurosurgical cure is not achieved in all cases; thus, medical treatment is vitally important for controlling hormone levels and eventually, tumor expansion. First-generation somatostatin receptor ligands (SRL) are recommended as a first-line medical therapy in all clinical guidelines, but biochemical control is only achieved in approximately 50% of patients or even less^3,4^. Furthermore, response to first-generation SRL can be partial, without achieving complete control of the hormonal excess^5^.

The delay in diagnosing acromegaly and finding the effective medical treatment negatively affects life expectancy and quality of life^6,7^. For this reason, personalized medicine would be a substantial improvement for acromegaly allowing physicians to assign the most appropriate treatment in terms of effectiveness for each case^8–10^. In a previous study, we confirmed that expression of E-cadherin in somatotropinomas is, so far, the best predictor of response to SRL^11,12^.

Different factors, such as age and sex^13,14^, radiologic information such as T2-weighted MRI signal intensity^15^, and histopathologic data such as granularity pattern^16,17^ are related to therapeutic outcomes. Tumor expression of *SSTR2* and other molecules have offered additional insights in relation to treatment response^11,18^, although some studies have shown controversial results^19^. Currently, the major drawback to transferring this approach to clinical practice is the overlapping of values of these markers between response categories which does not allow the definition of clear cut-offs. Moreover, it is difficult to account for many biological, clinical and molecular variables with small but added effects in the response to first-generation SRL. Using data mining, a modality of mathematical analysis allowing efficient subclassification of heterogeneous populations, such as those of GH-secreting tumors^20^, it is potentially possible to elicit different combinations of molecular markers expressed in somatotropinomas with predictive value. Since no single form of classification is appropriate for all data sets, a large toolkit of classification algorithms have been developed through the years (linear regression, logistic regression and naïve Bayes, among others)^21,22^. The underlying concept of this study is that applying data mining techniques by combination of the already discovered biomarkers of response to SRL and patient clinical phenotype we would achieve a better stratification of the patients than using single markers. Accordingly, here we provide the preliminary results of a proof-of-concept study in which combined data are analysed through artificial intelligence methods to identify high accuracy classifiers of first-generation SRL response categories.

## Methods

### Patients

This study is an in-depth statistical analysis of data generated in a previous study^11^ which included seventy-one acromegaly patients from the REMAH cohort^23^ who had undergone pituitary surgery and had tissue availability. Samples of somatotropinomas were obtained consecutively from surgeries at 26 Spanish tertiary centers, reflecting the daily practice of acromegaly management. Fifty-one acromegaly cases (51% females, mean age 45.3 ± 13y) received SRL treatment before surgery while the remaining 20 patients did not (51% females, mean age 44.6 ± 13 y). All patients were treated with SRL (octreotide or lanreotide) because of disease persistence after neurosurgery for at least 6 months under maximal effective therapeutic doses according to IGF1 values. SRL response was categorized as complete responders (CR), partial (PR), or non-responders (NR) if IGF1 was normal, between > 2 < 3 SDS, or > 3 SDS IGF1, respectively, as previously described^15^.

The tumors were macroadenomas in 79% of cases, 19% causing visual alterations and 28% hypopituitarism before surgery; 37.5% showed a hypointense T2 tumor signal. Mean BMI was 28 kg/m^2^ ± 4.8 SD; 28% presented diabetes, 32% dyslipidemia, and 35% hypertension.

The study was conducted in accordance with the principles of the Declaration of Helsinki/ International Conference on Harmonised Tripartite Guideline for Good Clinical Practice. The study was approved by the Germans Trias i Pujol Hospital Ethical Committee for Clinical Research (EO-11-080). All patients provided written informed consent.

### Clinical data

The categorical variables evaluated in this study were: *GNAS* mutation status, sex, presence of extrasellar growth and sinus invasion, T1 and T2 categorical MRI intensity signal, presurgical visual alterations, presurgical hypopituitarism, history of diabetes, high blood pressure, dyslipidaemia, cancer, cerebrovascular disease and cardiovascular disease. T1 and T2 categorical MRI intensity were assessed by each participating center as previously described by Potorac et al.^24^. Quantitative variables were: age, Body Mass Index (BMI), GH levels at diagnosis, GH levels after oral glucose overload at diagnosis, IGF1 diagnostic values, time under SRL therapy and tumor maximum diameter (mm). IGF1 and GH levels were measured in each center. IGF1 index at diagnosis was calculated by dividing each serum IGF-1 value by the upper limit of reference range for IGF1.

Regarding hormonal measurements, blood samples were collected from patients at baseline and at different follow-up times after an overnight fast. Serum IGF1 was measured by two different methods (Immunotech IGF1 kit; Immunotech-Beckman, Marseille, France and Diagnostic Systems Laboratories, Webster, Texas, USA) and normalized for comparisons by expressing SD values^11,15^.

### Molecular data

We used the relative gene expression data (the expression of every gene was assessed by RT-qPCR using Taqman assays and calculated relative to the expression of three reference genes) and mutational data obtained in our recent study^11^. Only one pediatric case harboured a mutation on the AIP gene and was excluded from the study.

### Biomarker data mining analyses

The molecular and clinical data of the acromegaly patients included in our recently published work^11^ were used. The novelty is the methodology for establishing algorithms and the generation of cut-off values, not previously published for the combined clinical and molecular determinants of acromegaly therapeutic response. First, an independence analysis between categorical variables and SRL response categories was performed by means of a Pearson’s Chi-squared test to identify dependencies. Evaluation of potential bias between centers was also performed.

For the quantitative variables a Kolmogorov–Smirnov test was applied to assess the normality of the samples. The differential behaviour of the variables studied according to SRL response groups was analysed applying a Student's t-test, or a Wilcoxon-rank sum (Mann Whitney U) test, depending on the Gaussian or non-Gaussian distribution of the variable values, respectively.

**Figure 1:**
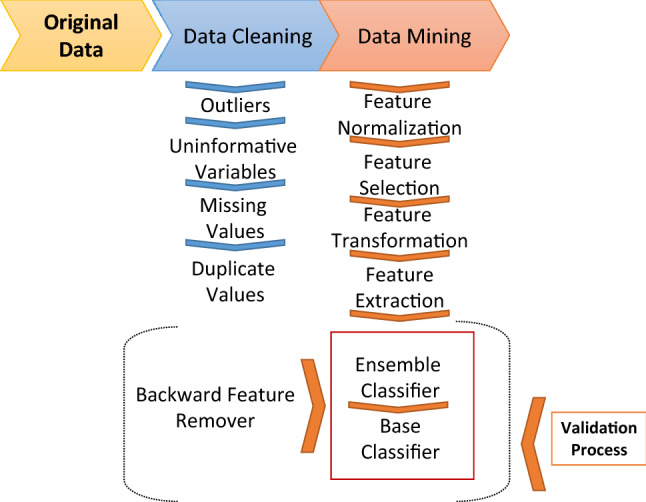
Biomarker data mining analyses procedure. First, a Data Cleaning process was performed to eliminate outliers, uninformative variables, missing values, and duplicate variables. Next, this new cleaned data set was used to train the model of the Data Mining process which is subdivided in different mathematical sub-processes: Feature Normalization, Feature Selection, Feature Transformation, Feature Extraction, Ensemble Classifier, Base Classifier, Backward Feature Removal and Validation. The Feature Normalization guarantees that the values of all variables are in the same range. The Feature Selection is applied to select the input variables that show the strongest relationship with the outcome. The Feature Transformation consists in mathematical transformations of the input data required for the Base Classifiers. It was not necessary to apply a Feature Extraction to reduce the number of random variables. Different algorithms generated different Base Classifiers with a good performance. Ensemble Classifiers were able to improve the performance of the Base Classifiers. Finally, the Validation process to estimate the accuracy of the predictive model was performed using the original database by several methods: 10-K fold and Leave-one-out.

**Figure 2:**
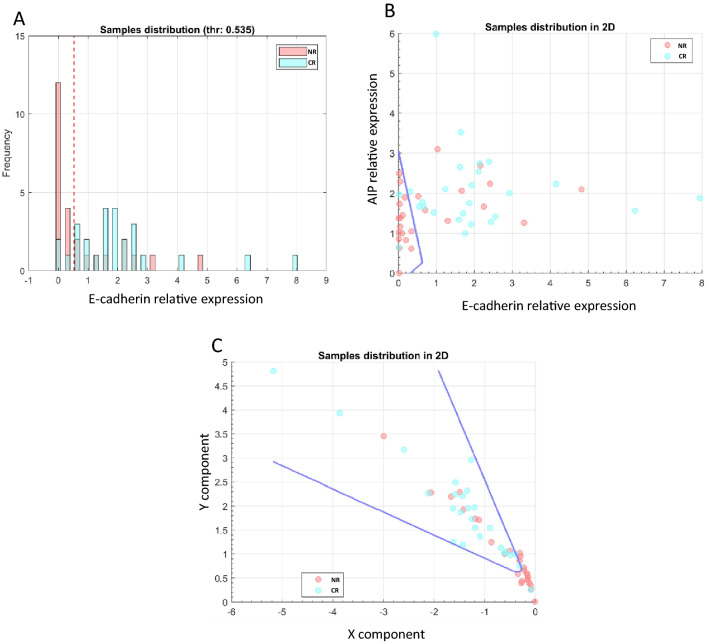
Representation of different possible models resulting from the data mining analysis in the whole cohort. (**A**) Sampling distribution graph representing the distribution of CR and NR patients for E-cadherin expression. When the classifier contains only one variable we used a variable brute force technique. The discriminant function is a constant that is determined as the threshold value that separates samples from the two groups with the best accuracy (marked by dotted red line). (**B**) Sampling distribution graph in 2D representing the distribution of CR and NR patients for the expression of *AIP* and E-cadherin. The blue line is the mathematical function defined by the values of the classifier, a mathematical function that separates NR from CR patients. As this classifier is composed of two variables, each dimension of the graph stands for one variable. The variables were selected by the Lasso method and the model performed according to Multilayer perceptron (MLP) methodology. (**C**) Sampling distribution graph in 2D representing the distribution of CR and NR patients for the expression of *SSTR2*, E-cadherin and *AIP*. As this classifier is composed of more than two variables, each dimension of the grafh stands for the the two main components after performing a principal component analysis (PCA). The blue line is the mathematical funtion that separates CR from NR patients. The variables were selected by the Wilcoxon method and the model performed according to Multilayer perceptron (MLP) methodology.

Data Mining strategy was applied by Anaxomics S.L. (http://www.anaxomics.com) to identify the best classifiers (Fig. 1)^25,26^ among quantitative variables. In order to add the information of the categorical data to the models, we divided the samples according to a categorical variable in what it is called “fragmented population”, for example, biological sex, and applied all the data mining strategies to the obtained subsets. This procedure was applied to different categorical variables. The fragmentation of population deconstructs the heterogeneity to overcome molecular differences and reduce statistical noise that is not due to SRL response. mRNA expression levels are treated as continuous variables in the models. First, a Data Cleaning process was performed to eliminate outliers (values > 3 times the standard deviation of the rest of values), uninformative variables (not considered because the values for all the samples are the same or variables with 100% coincidence with the outcome of the analysis), missing values, and duplicate variables. Next, this new cleaned data set was used to train the model of the data mining process. All the variables of the data set were individually evaluated for their capability as classifiers, in the whole and the categorical variable-fragmented populations. Missing data was not imputed in the classifiers. When the classifier contained only one variable, the discriminant function was a constant that was determined as the threshold value that separated samples from different groups with the best accuracy (Fig. 2A). The threshold value was determined iteratively and a cross-validation (10-K fold) protocol was performed. In contrast, when the classifier contained two or more independent variables, the discriminant function was generated by applying Data Science approaches that identified the best classifiers (Fig. 2B,C), and thus, the threshold could be single, double or a polynomial threshold line. This process was subdivided in different mathematical sub-processes: Feature Normalization, Feature Selection,

Feature Transformation, Feature Extraction, Ensemble Classifier, Base Classifier, Backward Feature Removal and Validation (Fig. 1). By means of artificial intelligence (AI) procedures, different mathematical algorithm approaches previously published were explored for each sub-process, allowing an exhaustive exploitation of the data (Table 1). In the present study the Feature Normalization determined that the values of all the variables were in the adequate range for the analysis, thus no further method of normalization was required. It was not necessary to apply a Feature Extraction to reduce the number of random variables. Different algorithms generated different classifiers. Since our goal was the prediction of SRL response for an individual case, we wanted to estimate how accurately a predictive model would perform in clinical practice. In order to flag selection bias or overfitting in our models, we used cross-validation techniques for assessing how the model would generalize to an independent data set. We confronted the model obtained with a subset of training data with the test data using a 10-K fold strategy. Therefore, we obtain a more exact estimation of the accuracy of the model taking the average of all the accuracy estimations obtained after each iteration. We used the accuracy (ACC) as the simplest parameter for evaluating the model, being the proportion of correct predictions (both true positives and true negatives) among the total number of samples. Accuracy levels are referred in these terms: accuracy 100–95%, excellent; 95%-80%, very good; 80%-70%, good; below 70%, to be improved.

**Table 1:** Mathematical methods explored during the different processes included in the Data Mining strategy.

Sub-process	Algorithm	References
Backward removal features	Backward elimination	^27^
Base classifier	Elastic net	^28^
	K-nearest neighbors (K-NN)	^29^
	Boosted Generalized Additive Models (B-GAM)	^30^
	Tree	^31^
	Support vector machine (SVM)	^32^
	Multilayer perceptron (MLP)	^33^
	MLP ensemble	^33^
	Linear search	^21^
	Linear regression	^21^
	Quadratic	^21^
	Random linear	^21^
	Generalized linear model binomial	^22^
	Ridge regression	^34^
	Naïve bayes	^35^
	Lasso regression	^36^
	Radial basis function (RBF)	^37^
Cost function	Accuracy	^38^
	Balanced accuracy	^38^
	Balanced cost matrix	^38^
	Cost matrix	^38^
	F1 score	^38^
	Matthews correlation coefficient (MCC)	^39^
	Area Under Curve (AUC)	^40^
Dimensionality reduction	Principal component analysis (PCA)	^41^
	T-distributed Stochastic Neighbor Embedding (t-SNE)	^42^
	Multidimensional scaling (MDS)	^43^
	Hessian locally linear embedding (HLLE)	^44^
	Isomap	^45^
	Latent Dirichlet allocation (LDA)	^46^
	Locally linear embedding (LLE)	^47^
	Sammon projection	^48^
	LandMark ISOMAP (L-ISOMAP)	^49^
	Laplacian	^50^
	Gaussian process latent variable model (GPLVM)	^51^
	Kernel PCA	^52^
	Independent component analysis (ICA)	^53^
	Non-negative matrix factorization (NMF)	^54^
	Factor analysis	^55^
	Probabilistic principal component analysis (PPCA)	^56^
	Local tangent space alignment (LTSA)	^57^
Ensemble classifier	Bootstrap	^58^
	Bootstrap respecting prevalence	^58^
	Balanced bootstrap	^58^
Ensemble method	Bootstrap	^59^
	Bootstrap respecting prevalence	^59^
	Balanced bootstrap	^59^
Feature selection	K-nearest neighbors (K-NN)	^29^
	Receiver operating characteristic (ROC)	^60^
	Bhattacharyya	^61^
	Ridge regression	^61^
	Wilcoxon	^62^
	Wilcoxon + correlation	^62^
	minimum Redundancy Maximum Relevance (mRMR) Mean discretized	^63^
	Boolean balanced three-valued logic rules	^64^
	Sequential floating forward selection (SFFS)	^65^
	Support vector machines recursive feature elimination (SVM-RFE)	^66^
	Random forest	^67^
	Chow-Liu	^68^
	Simple regression	^21^
	Relieff	^69^
	Random generalized linear model	^22^
	One variable brute force	^70^
	Bhattacharyya + Correlation	^71^
	Entropy	^71^
	Entropy + Correlation	^71^
	Mattest	^71^
	T-test	^71^
	T-test + Correlation	^71^
	minimum Redundancy Maximum Relevance (mRMR)	^72^
	Lasso	^36^
	Elastic net	^73^
	Double Cross-Validation regression	^74^
Feature transformation	Sigmoid	^71^
	Gaussian: the value used is the value obtained after being submitted to a Gaussian function	
	No value transformation	
	The value used is the original value multiplied by itself	
	The value used is the square root of the original value	
Multiclass classifier	Generalized coding	^71^
	One versus all (OVA) binary classified applied	
	One versus one (OVO) binary classifiers applied	
Normalization	Sigmoidal mean variance	^71^
	Trimmed mean variance	^71^
	Mean variance	
	Median dispersion	
	Min Max: each value is divided by the difference between the maximum and the minimum value	
	Winsorizing mean variance	
Validation	Bootstrap	^75^
	K-Fold	^76^
	LeaveOneOut (LOO)	^71^

## Results

### Phenotypical characterization according to first-generation SRL response

A phenotypical characterization was performed according to SRL response which showed that SRL resistance was strongly associated with tumor extrasellar extension (Pearson χ^2^ p‐value: 0.004) as shown in Table 2. Furthermore, NR patients presented more sinus invasion and hypopituitarism before surgery in contrast to CR or PR (Pearson χ^2^ p‐value: 0.05 and 0.01, respectively). However, it is debatable whether the association of hypopituitarism is of clinical significance since we would have expected a progressive behavior from CR to NR, thus with a potential association of NR with hypopituitarism which may have been related with a larger and more destructive adenoma rather than a marked difference in the PR group.

**Table 2:** Clinical categorical variables related to SRL response.

	Group	SRL response^a^			Pearson χ2 p-value^b^
		CR	PR	NR	
Presurgical hypopituitarism	Yes	42%	15%	55%	**0.01**
	No	68%	85%	45%	
Presurgical visual alterations	Yes	13%	27%	19%	0.62
	No	87%	73%	81%	
T2 signal intensity	Hypointense	31%	22%	36%	0.90
	Isointense	38%	56%	36%	
	Hyperintense	31%	22%	28%	
T1 signal intensity	Hypointense	61%	40%	53%	0.75
	Isointense	39%	50%	38%	
	Hyperintense	0%	10%	8%	
Gender	Male	46%	35%	62%	0.07
	Female	54%	65%	38%	
GNAS mutation	Mutated	29%	38%	36%	0.83
	WT	71%	62%	64%	
Sinus Invasion	Yes	22%	35%	59%	0.05
	No	78%	65%	41%	
Extrasellar growth	Yes	48%	60%	95%	**0.004**
	No	52%	40%	5%	

^a^SRL response columns indicate the percentage of patients with CR, PR, or NR dictated by the presence of absence of the clinical condition.

^b^Pearson χ2 p-values are shown. Statistically significant values (p-value < 0.05) are reported in bold.

Additionally, differences in the value of quantitative clinical variables according to SRL response categories were evaluated for the studied comparisons and the results are displayed in Table 3. High BMI and IGF1 levels at diagnosis were associated with NR patients.

**Table 3:** Clinical numerical variables showing differences between the evaluated comparisons.

Variable	CR + PR vs NR		CR vs NR		PR vs NR		CR vs PR	
	p-value	Log2FC	p-value	Log2FC	p-value	Log2FC	p-value	Log2FC
IGF1 diagnosis	**0.035**	**− 0.33**	**0.007**	**− 0.47**	0.722	**− **0.16	*0.081*	**− ***0.31*
IGF1 index diagnosis	*0.051*	**− ***0.41*	*0.086*	**− ***0.39*	*0.063*	**− ***0.43*	0.838	0.04
GH diagnosis	0.590	1.04	0.134	0.94	0.429	1.17	0.134	**− **0.22
GH after OGTT	0.622	1.27	0.728	1.29	0.633	1.25	0.941	0.03
BMI diagnosis	*0.094*	**− ***0.13*	**0.044**	**− 0.17**	0.452	**− **0.07	0.316	**− **0.10
Maximum diameter	0.178	**− **0.27	*0.092*	**− ***0.35*	0.532	**− **0.16	0.708	**− **0.19
Age diagnosis	0.197	0.14	0.272	0.13	0.802	**− **0.03	0.276	0.16

The clinical numerical variables that were tested: IGF1 levels measured at diagnosis in each center, IGF1 index at diagnosis, GH levels measured at diagnosis in each center, GH levels measured after a 75 g oral glucose load (OGTT), BMI (Body Mass Index) at diagnosis, maximum tumor diameter in the MRI measured in each center and the age of the patient at diagnosis. T-test or Wilcoxon-test p-values are shown. Statistically significant values (p-value < 0.05) are reported in bold, and p-value < 0.1 in italic Log2FC: Log2 Fold Change.

### Algorithms classifying SRL response in acromegaly patients

The in-depth statistical exploration of the data generated in our previous paper^11^ allowed to formulate several algorithms for the discrimination of patients regarding SRL response (cross‐validated p‐value < 0.05); those displaying the highest accuracy are shown in Table 4. All the significant predictive models are presented in Supplementary Tables. The strongest and most accurate single predictive biomarker for SRL response was E-cadherin, as it was the only marker discriminating between 3 of the 4 comparisons categories evaluated: (1) CR vs PR accuracy 65.8% at cut-off values of 0.513 and 0.007; (2) CR vs NR accuracy 73.1% at cut-off value 0.535; (3) CR + PR vs NR accuracy 62.6% at cut-off values of 0.348 and 0.013. Moreover, E-cadherin was also found in many of the dual and triad panels obtained by the analysis. After E-cadherin, the most frequent contributor to enhance classification power was *SSTR2*. The combination of E-cadherin and *SSTR2* increased the accuracy by 6–7% more than E-cadherin alone. The addition of *AIP*^77^ or In1-GHRL^78^ showed a moderate enhancement of the classification power, reaching 75% of accuracy. Finally, adding *PEBP*^79^ displayed nearly a 70% accuracy at cut-off 15.56, specifically in the discrimination between CR and PR.

**Table 4:** Best classifiers in the whole cohort.

Evaluated comparison	Panel of classifiers	ACC	p-value
CR + PR vs NR	E-cadherin	62.61%	0.027
	*GHRL*	67.26%	0.002
	*SSTR2* + E-cadherin	69.95%	0.001
CR vs NR	*DRD2* long isoform	69.23%	0.006
	E-cadherin	73.08%	0.001
	*SSTR2* + E-cadherin + *AIP*	75.00%	< 0.001
	*SSTR2* + E-cadherin + *IN1GHRL*	75.00%	< 0.001
PR vs NR	*SSTR2* + Ki-67	67.87%	0.02
	*SSTR2* + *SSTR5* + *ARRB1*	69.68%	0.004
CR vs PR	E-cadherin	65.84%	0.028
	*PEBP1*	69.68%	0.004

All individual classifiers and those panels with 2 or 3 classifiers that display an improvement in accuracy are presented in this table. ACC: Accuracy.

For those panels including more than one marker, in pairs or triads, cut-off values showed dynamic values (the values change with respect the variables of the model as a function because the variables are interdependent) as shown in Fig. 2B,C.

### Fragmented population analysis achieves higher predictive accuracy

For analysis purposes, the cohort was subsequently segregated according to different clinical and biological variables, such as sex, extrasellar growth of the tumor, radiological sinus invasion, the mutational status of *GNAS*, T2 hypointense signal^80^ and presurgical SRL treatment. The fragmented population studied is detailed in Supplementary Table 1.

The analysis provided multiple models depending on the core variable used in the fragmentation. The best models for every clinical scenario are shown in Table 5. Overall, the algorithms generated achieved a much higher cross‐validated accuracy in the fragmented rather than in the whole cohort for prediction of SRL response, as detailed in Supplementary Tables.

**Table 5:** Best classifiers in patients with or without SRL presurgical treatment, extrasellar growth, sinus invasion, biological sex and *GNAS* mutational status.

Fragmenting condition	Evaluated comparison	Fragmented population N^a^	Best panel of classifiers	ACC	p-value
A. SRL presurgical treatement	CR + PR vs NR	No (9 vs 7)	*PLAGL1* + *PEBP1* + E-cadherin	88.89%	0.003
		Yes (33 vs 19)	*SSTR5* + *DRD2* long isoform + E-cadherin	70.65%	0.001
	CR vs NR	No (6 vs 7)	Age + *SSTR2* + E-cadherin	100.00%	5.83E−04
		Yes (20 vs 19)	*PLAGL1* + *IN1GHRL* + E-cadherin	76.97%	9.43E−04
	PR vs NR	No (3 vs 7)	Not found	–	–
		Yes (13 vs 19)	*SSTR5* + *PEBP1*	74.29%	0.003
	CR vs PR	No (6 vs 3)	*SSTR2* + E-cadherin	100%	0.012
		Yes (20 vs 13)	*PEBP1* + *IN1GHRL*	76.82%	4.02E−04
B. Extrasellar growth	CR + PR vs NR	No (18 vs 1)	Not found	–	–
		Yes (20 vs 19)	*GHRL*	71.32%	0.005
	CR vs NR	No (12 vs 1)	Not found	–	–
		Yes (11 vs 19)	Not found	–	–
	PR vs NR	No (6 vs 1)	Not found	–	–
		Yes (9 vs 19)	Not found	–	–
	CR vs PR	No (12 vs 6)	*SSTR5* + *PEBP1*	87.50%	0.004
		Yes (11 vs 9)	*SSTR5* + *IN1GHRL* + E-cadherin	79.80%	0.012
C. Sinus Invasion	CR + PR vs NR	No (26 vs 7)	Not found	–	–
		Yes (12 vs 10)	*AIP*	77.50%	0.015
	CR vs NR	No (18 vs 7)	*SSTR2* + *ARRB1* + *KLK10*	81.75%	0.007
		Yes (5 vs 10)	*PEBP1* + *AIP* + *IN1GHRL*	85.00%	0.017
	PR vs NR	No (8 vs 7)	Ki-67 + *IN1GHRL*	85.71%	0.007
		Yes (7 vs 10)	Not found	–	–
	CR vs PR	No (18 vs 8)	*SSTR2* + *IN1GHRL* + *KLK10*	86.61%	0.009
		Yes (5 vs 7)	Not found	–	–
D. Gender	CR + PR vs NR	Female (25 vs 10)	*PEBP1* + *GHRL*	73.78%	0.007
		Male (18 vs 16)	Age + E-cadherin	80.83%	0.001
	CR vs NR	Female (14 vs 10)	*PEBP1* + E-cadherin + *AIP*	79.76%	0.005
		Male (12 vs 16)	Age + *PLAGL1* + E-cadherin	85.45%	4.91E−04
	PR vs NR	Female (11 vs 10)	Not found	–	–
		Male (6 vs 16)	*SSTR2* + *PLAGL1* + *GHRL/ARRB1*	85.35%	0.003
	CR vs PR	Female (14 vs 11)	*SSTR2* + *PEBP1*	74.68%	0.016
		Male (12 vs 6)	*DRD2* short and long isoform + E-cadherin	80.00%	0.018
E. *GNAS* mutational status	CR + PR vs NR	WT (19 vs 14)	*SSTR2* + *DRD2* long isoform + *ARRB1*	77.07%	0.003
		Mutated (10 vs 5)	Not found	–	–
	CR vs NR	WT (10 vs 14)	Not found	–	–
		Mutated (5 vs 5)	*PLAGL1* + E-cadherin + Ki-67	90.00%	0.024
	PR vs NR	WT (9 vs 14)	*SSTR5* + *ARRB1*	72.22%	0.014
		Mutated (5 vs 5)	Not found	–	–
	CR vs PR	WT (10 vs 9)	*PEBP1* + E-cadherin	84.44%	0.004
		Mutated (5 vs 5)	Not found	–	–
F. Hypointense T2 signaling	CR + PR vs NR	NO HYPO (23 vs 15)	*SSTR3* + *ARRB1* + *AIP*	74.18%	0.008
		HYPO (14 vs 8)	*DRD2* short isoform + Ki-67	75.00%	0.040
	CR vs NR	NO HYPO (13 vs 15)	*SSTR3* + *SSTR2* + Ki-67	88.46%	8,75E−05
		HYPO (9 vs 8)	E-cadherin	87.50%	0.003
	PR vs NR	NO HYPO (10 vs 15)	Age + *DRD2* short isoform + *PEBP1*	76.79%	0.022
		HYPO (5 vs 8)	Not found	–	–
	CR vs PR	NO HYPO (10 vs 9)	*DRD2* short isoform + *KLK10*	85.04%	0.001
		HYPO (5 vs 5)	Not found	–	–

For each subgroup, the best panel/s of classifiers (with accuracy higher than the maximal one achieved by the classifiers using the whole cohort without fragmentation) in each comparison are shown. ^a^The third column refers to the condition in the first column. *ACC* Accuracy.

### Decision tree therapeutic algorithms based on mathematical modelling

**Figure 3:**
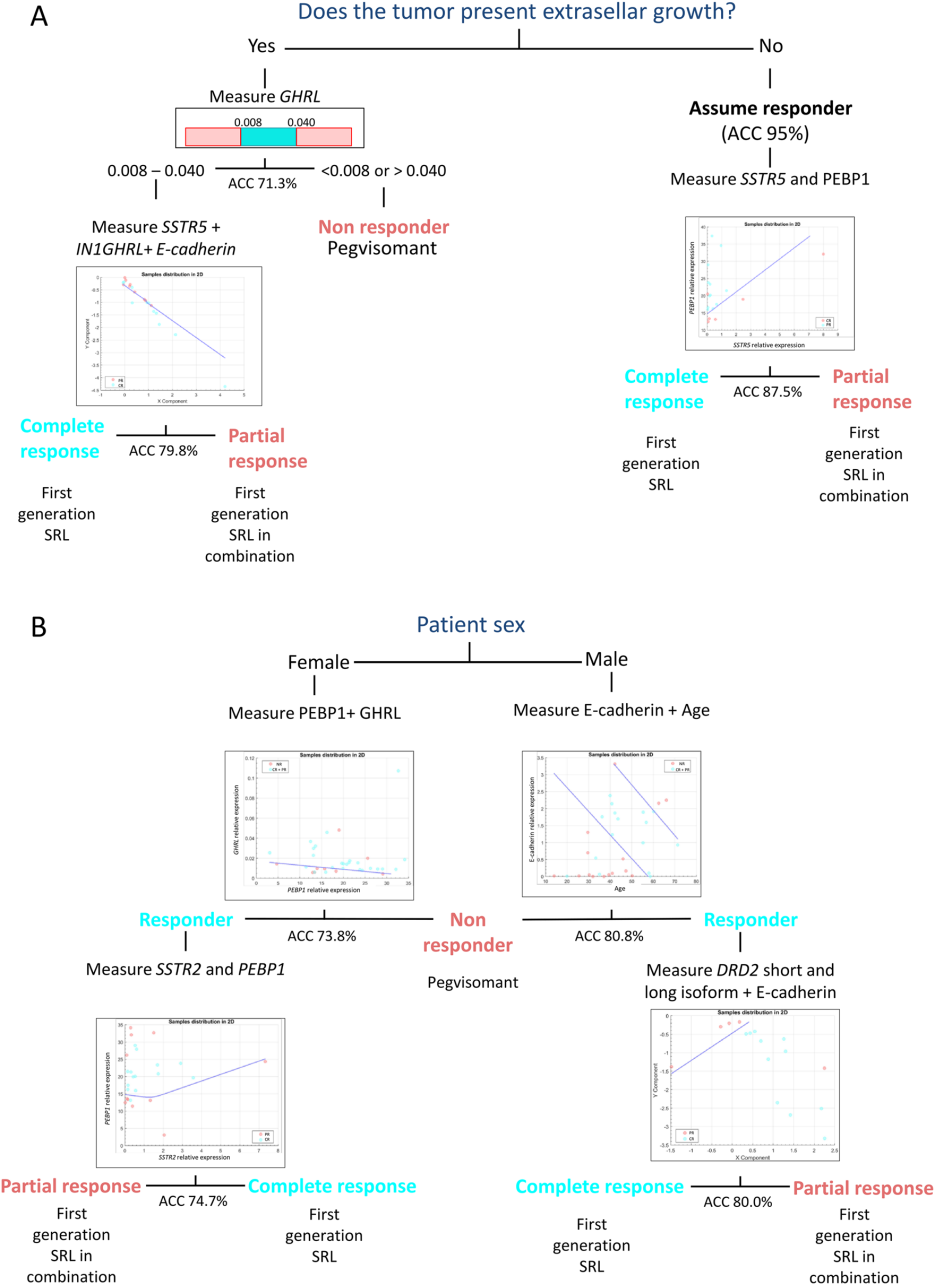
Best therapeutic tree decision algorithms based on mathematical modelling. (**A**) Decision tree to determine the first line drug for a given acromegaly patient based on the extrasellar tumor growth and molecular information. A patient without extrasellar growth is automatically classified as CR/PR without performing any molecular analysis (NR category is discarded with an accuracy of 95%). Then, by measuring the gene expression of *SSTR5* and *PEBP1* a clinician would be able to assign the right treatment with an accuracy of 87.5%. If the tumor has extrasellar growth, the gene expression of *GHRL* should be measured. If levels are < 0.008 or > 0.04, the patient is classified as NR with an accuracy of 71.3%, while if levels are between 0.008 and 0.04, the patient is classified as CR/PR. Then, by measuring the gene expression of *SSTR5, IN1GHRL* and E-cadherin a clinician would be able to assign the right treatment with an accuracy of 79.8%. When classifiers are composed of more than one variable (e.g. *SSTR5* and *PEBP1 or SSTR5, IN1GHRL* and E-cadherin), the distribution of CR and PR patients is defined by a mathematical function (the blue line in the scatterplots) that separates CR from PR patients (blue and pink dots in the scatter plots, respectively). The details of the scatter plots and the mathematical models can be found in the Supplementary Figures S1-S3. (**B**) Decision tree exploiting molecular differences according to sex to accurately treat an acromegaly patient. If the patient is a male, the expression of E-cadherin should be measured and together with age it would be able to classify the patient as NR with an accuracy of 80.8%. If it is classified as CR/PR, the expression of the short and long DRD2 isoforms should be analyzed and together with E-cadherin it would be able to assign the right treatment with an accuracy of 80.0%. If the patient is a female, the expression of *PEBP1* and *GHRL* should be measured and this will allow to classify the patient as NR with an accuracy of 73.8%. If it is classified as CR/PR, the expression of the short and long DRD2 isoform should be analyzed and together with E-cadherin it would allow to assign the right treatment with an accuracy of 74.7%. The details of the scatter plots and the mathematical models can be found in the Supplementary Figures S4-S7. *ACC* Accuracy, *CR* complete responder, *PR* partial responder, *NR* non-responder.

The present analyses allow the development of decision trees that may be used in clinical practice for individual patients. Two trees were formulated. The first one is based on the extrasellar tumor growth and different molecular biomarkers (Fig. 3A). A patient without extrasellar growth is discarded as NR with an accuracy of 95%, and for distinction between CR and PR, the measurement of *PEBP1* and *SSTR5* allows to achieve an accuracy of 87.5%. When tumor extrasellar growth is present, the decision tree segregates NR patients from responders (CR and PR) using levels of *GHRL* expression with an accuracy of 71.3%. To differentiate between CR and PR, measurement of *SSTR5*, In1-GHRL and E-cadherin leads to an accuracy of 79.8%. A second tree based on the patient’s sex showed an accuracy of 73.8–80.8% to distinguish between NR, CR and PR patients, being higher for men than for women (Fig. 3B).

Both algorithms show a high accuracy to identify NR patients (accuracy ranging from 71.3 to 95%) which is particularly important since NR are the patients that suffer the largest delay using the current fixed sequential therapeutic decision chart. In all cases, measuring the expression of one or two molecules would be enough to define this type of patient response. The accuracy to distinguish between CR and PR patients is lower except for patients without extrasellar growth, thus we recommend the use of these algorithms specially to identify NR patients. When models are combined, the accuracies of the different steps should be multiplied to obtain the total final accuracy. Detailed mathematical features of the models can be found in Supplementary Figures S1-7.

## Discussion

General findings in our cohort included a substantial association between first-generation SRL response and invasive tumors. BMI and IGF1 basal levels were also slightly associated with SRL response. Although high BMI used to be associated with acromegaly condition^81^, it is the first time that this association has been also identified regarding SRL response. Also, molecular differences match with the sexual dimorphism of SRL response^82^. In particular, *PEBP1* was associated with the prediction of SRL response in women more than in men, as previously reported^79^. Moreover, age, which has also been considered as a SRL response factor^83^, seems to be more important in men. Furthermore, as we firstly^11^ reported, the hypointense T2 MRI signal was associated with a better SRL response, also confirmed by others^84^. In our cohort, non T2-hypointense tumors showed less heterogeneity allowing a better classification by AI procedures. Interestingly, *SSTR3* contributed to classify the T2-hypointense tumors while it was not associated with any other clinical feature.

Nonetheless, single markers are not powerful enough to achieve a highly accurate and discriminative capacity of first-generation SRL response categorization in such heterogeneous disease as acromegaly. Our data definitely confirm that E-cadherin is one of the most powerful markers of SRL response prediction, as initially described by Fougner et al.^85^. In our analysis *SSTR2*, although being a cardinal biomarker for developing a predictive algorithm, was insufficient as a single marker tool of SRL response prediction. The variability in the ability of *SSTR2* to predict SRL response has been reported in different studies. Some authors found no statistical differences between *SSTR2* and SRL response^19^ while others did^86,87^. Wildemberg et al. assessed the performance of *SSTR2* as a marker of SRL response and found a sensitivity of 100% and specificity of 38%^88^, which represent a better sensitivity but a worse specificity compared to what we previously found (60% and 75%, respectively)^11^. These differences may be due to the use of different methodologies to quantify *SSTR2*, to the criteria applied to categorize patient’s response or to biological differences between the cohorts, as these tumors are highly heterogeneous.

Most of the molecules that previously emerged from classical candidate gene approach as potential biomarkers of response to SRL are fairly represented in the algorithms and decision trees obtained in our analyses using data mining. Thus, from the different molecules previously reported as single markers: E-cadherin, *SSTR2, PEBP1*, *GHRL* and In-1-GHRL, and *AIP* are those that contribute -with different combinations at individual level- more robustly to the generation of decision trees and models in our cohort. Regarding *AIP*, although mutations in that gene are the most frequent germline mutations in somatotropinomas^89^ and are associated with poorly response to first generation SRL response, our cohort did not include any AIP-mutated case. Instead, we analyzed *AIP* expression since *AIP* levels have been also related to SRL resistance^90,91^.

To date, the best single marker is just able to predict with an accuracy not higher than 70%. In our study we were able to obtain accuracies that were above 70% and in some cases were ranging from 80 to 100% depending on the algorithm, thus one of the conclusions of our work is that in the future, acromegaly patients with specific characteristics will probably require specific decision trees obtained from enriched large cohorts. In this regard the present study is a preliminary work with internal validation procedures but awaiting of external validation with other similar cohorts.

The other very important issue is the definition of the cut-off values for application to clinical practice; in the present study we have been able to define cut-off values for the different clinical scenarios which may be useful for clinical implementation. The cut-off values obtained are not precise numbers applicable to all patients but instead they are dynamic, interdependable values calculated from the formulated equations (the mathematical models) that change for every single patient according to his or her clinical characteristics and/or to the expression of the markers in the tumor. The mathematical models we present, once established, will be easy to use, provided that the necessary biological markers will be determined in the tumor tissue. This kind of model is already used in other medical specialties, such as oncology. We strongly believe that acromegaly is a disease that will benefit enormously from this type of model decision algorithm. First, because there is an increasing number of therapies available; so, the “trial and error” approach would be unethical and impractical in the near future. Secondly, although acromegaly is a chronic disease and usually not acutely life-threatening, modern medicine is focused on quality of life which is heavily impaired in acromegaly and achieving a fast biochemical control could improve it considerably. Moreover, patient-reported outcomes (PRO) are increasingly been considered as the gold standard and included in guidelines and decisions by policy makers. In this regard, to have the option of choosing the most appropriate treatment for a given patient is the aim of contemporary medicine.

The present study has some limitations, being the most important the relatively low number of cases, but our results provide a proof-of-concept for the use of data mining strategies in the management of acromegaly patients. Thus, a constraint for implementation of personalized medicine, whether derived from classic or novel methods, is the necessity of validation of the proposed algorithms with other cohorts. However, by using data mining, the intrinsic nature of the mathematical analysis performs a continuous internal validation process; despite this, an external validation by an international consortium, capable of establishing a large cohort of acromegaly patients would be essential, since a substantial bias remains when this methodology is applied to small data sets^92^. Nonetheless, a study performed in a Brazilian cohort found models with a very similar performance^93^. The mathematical modelling was very similar in both studies but the data used to construct the models were very different. The Brazilian cohort was larger, consisting of 153 patients in total, and the models were generated using demographic data (age and sex), biochemical data (GH and IGF1 levels at diagnosis and before SRL treatment) and immunohistochemical data (granulation pattern and immunoreactivity score of SSTR2 and SSTR5), but they did not include MRI information. On the other hand, while we used RT-qPCR to quantify the molecular biomarkers, they used immunohistochemistry, a more widely used technique easily found in most hospitals but whose results are particularly operator-dependent. Another difference lies in the categorization of SRL response. In the Brazilian study, they divided SRL response in two categories: CR and patients that do not achieve biochemical control with SRL (corresponding to the PR + NR patients of our classification). So, the aim of Wildemberg et al. was to identify CR, whereas our main goal was to discriminate NR from patients for those who SRL could be useful. In any case, the models from both studies still have some space of improving their performance in order to achieve accuracy at 95% level. Thus, the inclusion of other biomarkers not yet identified may certainly improve final obtained accuracy warranting further discovery investigation using omics approaches to complete all the molecular actors that may explain SRL response in an individual case at the molecular level. Finally, The use of RT-qPCR to measure the biomarkers may be a limitation since it requires specialized instruments not available in many centers; however, qPCR instrumentation and the use of qPCR-based tests are rapidly increasing in clinical laboratories, mainly because qPCR is a highly sensitive, specific and quantitative method, and it is a must in a specialized pituitary tertiary center as defined by the Pituitary Society^94^.

In spite of the limitations, our preliminary results provide a proof-of-concept for the use of data mining strategies to generate improved mathematical algorithms that allow to apply personalized medicine and select the most suitable medical treatment for each acromegaly patient.

## Supplementary Information

Supplementary Information 1.

Supplementary Information 2.

## Data Availability

The data that support the findings of this study are available on request from the corresponding authors. The data are not publicly available due to privacy and ethical restrictions.
