# Supplementary material for: Data mining analyses for precision medicine in acromegaly: a proof of concept
Source: Sci Rep. 2022 May 28;12:8979. doi: 10.1038/s41598-022-12955-2 (PMC9148300; doi:10.1038/s41598-022-12955-2)
Supplement: Supplementary file 1 — Supplementary Information 1. [file 41598_2022_12955_MOESM1_ESM.pdf]

Data mining analyses for precision medicine in acromegaly: a proof of concept

Joan Gil et al.

Supplementary Figures

	page
**Supplementary Figure S1**	2
**Supplementary Figure S2**	3
**Supplementary Figure S3**	4
**Supplementary Figure S4**	5
**Supplementary Figure S5**	6
**Supplementary Figure S6**	7
**Supplementary Figure S7**	8

Supplementary Figure S1

A

Selected Strategy

**Feature Selection Method**	One variable brute force
**Base classifier**	Optimal quadratic
**Ensemble**	*Not applied*
**Cost function**	Balanced accuracy
**Validation**	10 K-fold

B

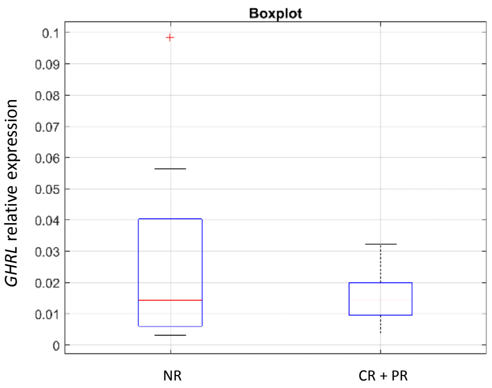

C

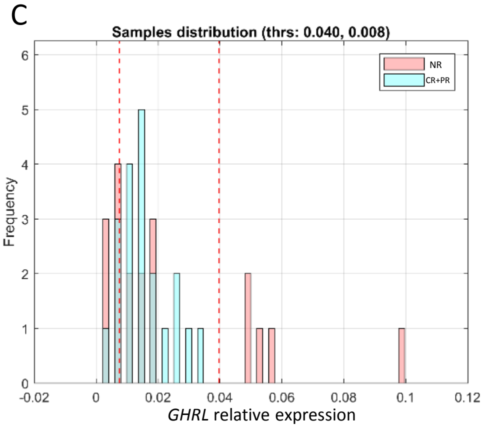

D

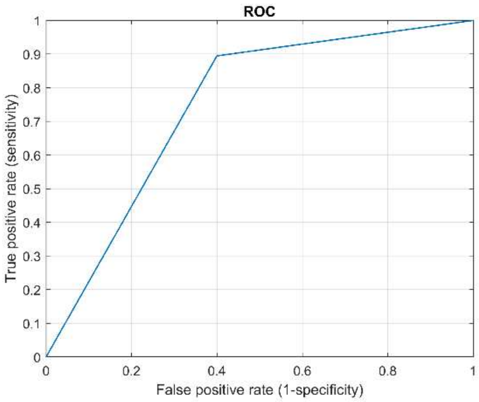

E

Samples separability test: Cross-validated

ACC	ACC p-value	TP	TN	FP	FN	PRE	SNS	SPC	Balanced ACC
71.79%	0.005	18	10	9	2	66.67	90.00	52.63	0.71

Samples separability test: without cross-validation

ACC	ACC p-value	TP	TN	FP	FN	PRE	SNS	SPC	Balanced ACC
76.92%	0.001	18	12	7	2	72.00	90.00	63.16	0.77

Supplementary Figure S1. *GHRL* model that discriminates between NR and CR+PR in patients with extrasellar growth. *GHRL* allowed the classification between NR and CR+PR in patients with extrasellar extension from our dataset. The details of the model subprocesses are presented in the table (A). The boxplot shows that there is not difference in the medians of *GHRL* expression (p=0.92) between NR and CR+PR patients but the distribution in each population is different (B), as we can clearly observe in the distribution graph of the samples (the dotted red lines indicates the best accuracy thresholds) (C). The Receiver Operating Characteristic (ROC) illustrates the performance of a our model as its discrimination threshold varies (D). Finally, the tables showing the performance in the sample separability tests allows for a clear evaluation of the model performing (E).
Abreviations: ACC (Accuracy), TP (True positives), TN (True negative), FP (False positive), FN (False Negative), PRE (Precision), SNS (Sensitivity), SPC (Specificity).

Supplementary Figure S2

A

Selected Strategy

**Feature Selection Method**	Lasso
**Base classifier**	Genealized linear method (Binomial)
**Ensemble**	*Not applied*
**Cost function**	Balanced accuracy
**Validation**	10 K-fold

B

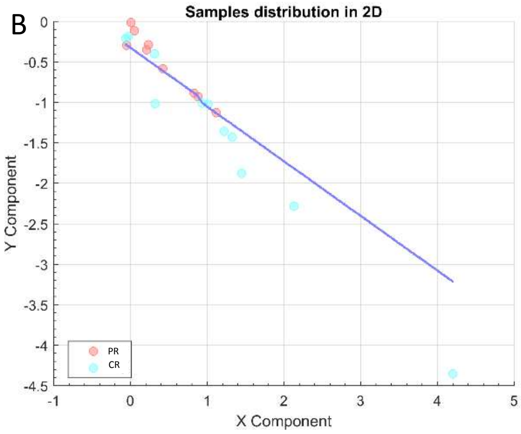

C

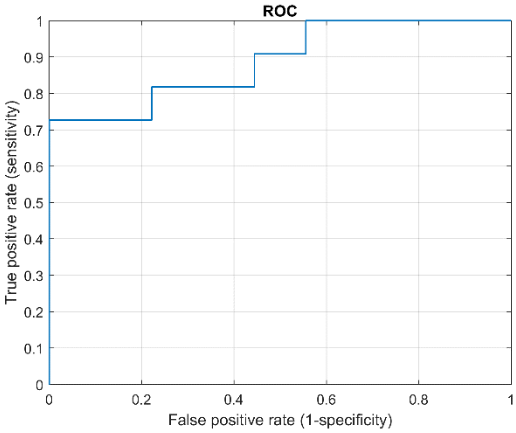

D

Samples separability test: Cross-validated

ACC	ACC p-value	TP	TN	FP	FN	PRE	SNS	SPC	Balanced ACC
80.00%	0.012	9	7	2	2	81.82	81.82	77.78	0.80

Samples separability test: without cross-validation

ACC	ACC p-value	TP	TN	FP	FN	PRE	SNS	SPC	Balanced ACC
75.00%	0.035	8	7	2	3	80.00	72.73	77.78	0.75

Supplementary Figure S2. E-cadherin + *SSTR5* + IN1-GHRL model that discriminates between CR and PR in patients with extrasellar growth. *E-cadherin, SSTR5* and *In1-GHRL* allowed the classification between PR and CR in patients with extrasellar extension in our dataset. The details of the model subprocesses are presented in the table (A). The graph represents the distribution of the samples in a 2D plot. The blue line is the mathematical function defined by the values of the classifier. X and Y components are obtained by means a Dimensionality Reduction Process (B). The Receiver Operating Characteristic (ROC) illustrates the performance of a our model as its discrimination threshold varies (C). Finally, the tables showing the performance in the sample separability tests allows for a clear evaluation of the model performing (D).
Abreviations: ACC (Accuracy), TP (True positives), TN (True negative), FP (False positive), FN (False Negative), PRE (Precision), SNS (Sensitivity), SPC (Specificity).

Supplementary Figure S3

A

Selected Strategy

**Feature Selection Method**	Random forest
**Base classifier**	Genealized linear method (Binomial)
**Ensemble**	*Not applied*
**Cost function**	Balanced accuracy
**Validation**	LeaveOneOut (LOO)

B

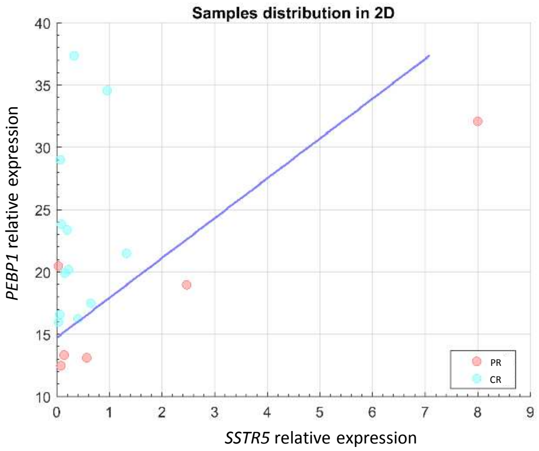

C

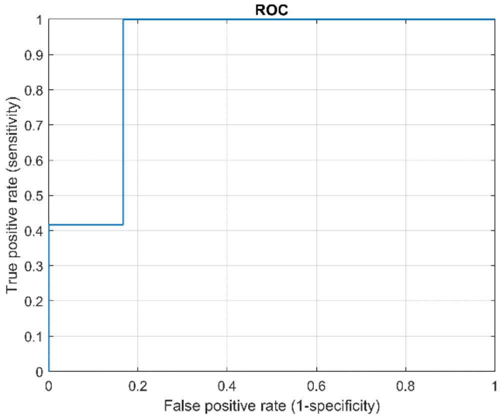

D

Samples separability test: Cross-validated

ACC	ACC p-value	TP	TN	FP	FN	PRE	SNS	SPC	Balanced ACC
88.89%	0.004	11	5	1	1	91.67	91.67	83.83	0.88

Samples separability test: without cross-validation

ACC	ACC p-value	TP	TN	FP	FN	PRE	SNS	SPC	Balanced ACC
94.44%	0.001	12	5	1	0	92.31	100.00	83.33	0.92

Supplementary Figure S3. *SSTR5* + *PEBP1* model that discriminates between CR and PR in patients without extrasellar growth. *SSTR5* and *PEBP1* allowed the classification between PR and CR in patients without extrasellar extension in our dataset. The details of the model subprocesses are presented in the table (A). The graph represents the distribution of the samples in a 2D plot. The blue line is the mathematical function defined by the values of the classifier (B). The Receiver Operating Characteristic (ROC) illustrates the performance of a our model as its discrimination threshold varies (C). Finally, the tables showing the performance in the sample separability tests allows for a clear evaluation of the model performing (D).

Abreviations: ACC (Accuracy), TP (True positives), TN (True negative), FP (False positive), FN (False Negative), PRE (Precision), SNS (Sensitivity), SPC (Specificity).

Supplementary Figure S4

A

Selected Strategy

Feature Selection Method	Relieff
**Base classifier**	Multilayer perceptron (MLP)
**Ensemble**	*Not applied*
**Cost function**	Balanced accuracy
**Validation**	10-K fold

B

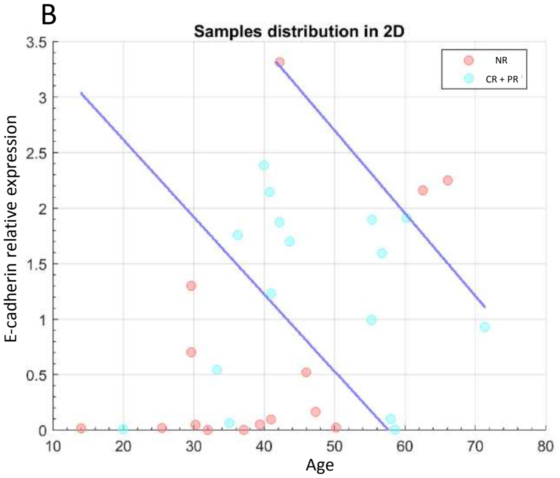

C

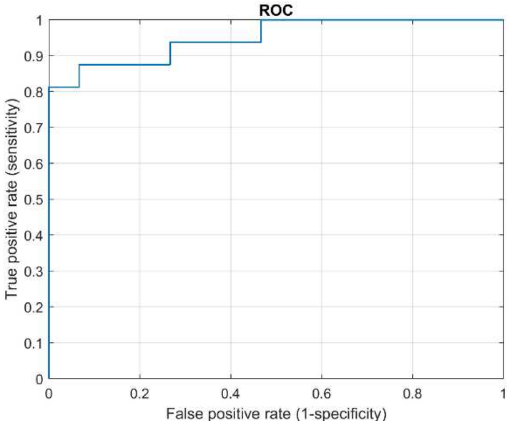

D

Samples separability test: Cross-validated

ACC	ACC p-value	TP	TN	FP	FN	PRE	SNS	SPC	Balanced ACC
80.65%	0.001	12	13	2	4	85.71	75.00	86.67	0.81

Samples separability test: without cross-validation

ACC	ACC p-value	TP	TN	FP	FN	PRE	SNS	SPC	Balanced ACC
90.32%	< 0.001	13	15	0	3	100.00	81.25	100.00	0.91

Supplementary Figure S4. Age + E-cadherin model that discriminates between NR and CR+PR in male patients. E-cadherin and the age of the patients allowed the classification between responders (CR + PR) and NR in male patients. The details of the model subprocesses are presented in the table (A). The graph represents the distribution of the samples in a 2D plot. The blue line is the mathematical function defined by the values of the classifier (B). The Receiver Operating Characteristic (ROC) illustrates the performance of a our model as its discrimination threshold varies (C). Finally, the tables showing the performance in the sample separability tests allows for a clear evaluation of the model performing (D).

Abreviations: ACC (Accuracy), TP (True positives), TN (True negative), FP (False positive), FN (False Negative), PRE (Precision), SNS (Sensitivity), SPC (Specificity).

Supplementary Figure S5

A

Selected Strategy

**Feature Selection Method**	Simple regression
**Base classifier**	Genealized linear method (Binomial)
**Ensemble**	*Not applied*
**Cost function**	Balanced accuracy
**Validation**	10 K-fold

B

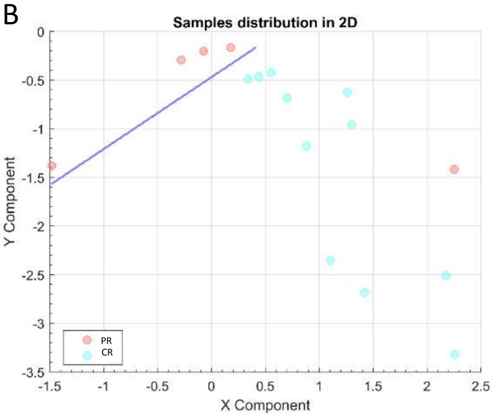

C

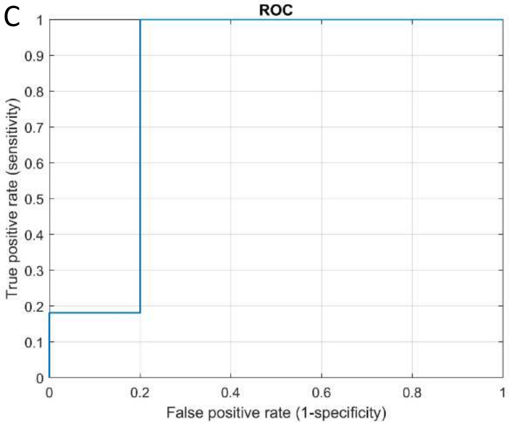

D

Samples separability test: Cross-validated

ACC	ACC p-value	TP	TN	FP	FN	PRE	SNS	SPC	Balanced ACC
87.50%	0.018	11	3	2	0	84.62	100.00	60,00	0.80

Samples separability test: without cross-validation

ACC	ACC p-value	TP	TN	FP	FN	PRE	SNS	SPC	Balanced ACC
93.75%	0.003	11	4	1	0	91.67	100.00	80.00	0.90

Supplementary Figure S5. E-cadherin + *DRD2* long and short isoform model that discriminates between CR and PR in male patients. *E-cadherin* and *DRD2* long and short isoform allowed the classification between PR and CR in male patients in our dataset. The details of the model subprocesses are presented in the table (A). The graph represents the distribution of the samples in a 2D plot. The blue line is the mathematical function defined by the values of the classifier. X and Y components are obtained by means a Dimensionality Reduction Process (B). The Receiver Operating Characteristic (ROC) illustrates the performance of a our model as its discrimination threshold varies (C). Finally, the tables showing the performance in the sample separability tests allows for a clear evaluation of the model performing (D).
Abreviations: ACC (Accuracy), TP (True positives), TN (True negative), FP (False positive), FN (False Negative), PRE (Precision), SNS (Sensitivity), SPC (Specificity).

Supplementary Figure S6

A

Selected Strategy

**Feature Selection Method**	Random generalized linear model
**Base classifier**	Multilayer perceptron (MLP)
**Ensemble**	*Not applied*
**Cost function**	Balanced accuracy
**Validation**	10 K-fold

B

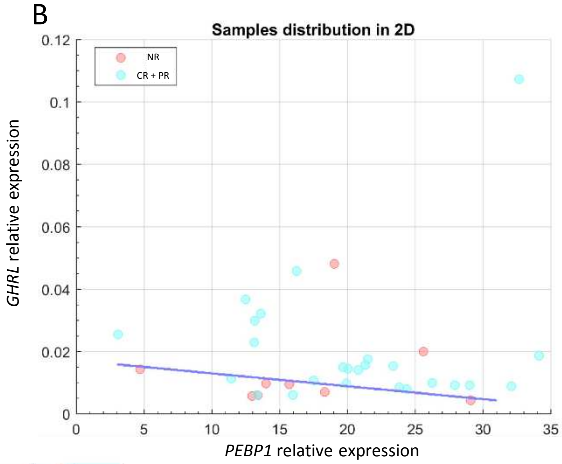

C

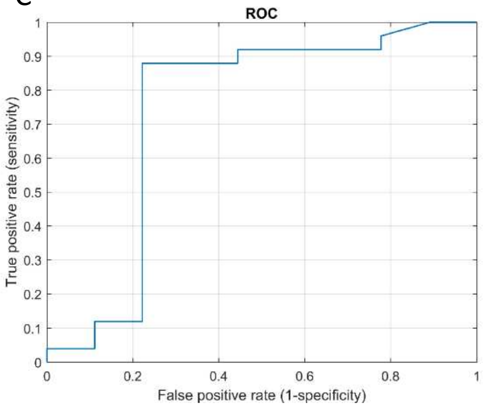

D

Samples separability test: Cross-validated

ACC	ACC p-value	TP	TN	FP	FN	PRE	SNS	SPC	Balanced ACC
82.35%	0.007	23	5	4	2	85.19	92.00	55.65	0.74

Samples separability test: without cross-validation

ACC	ACC p-value	TP	TN	FP	FN	PRE	SNS	SPC	Balanced ACC
85.29%	0.001	22	7	2	3	91.67	88.00	77.78	0.83

Supplementary Figure S6. *GHRL* + *PEBP1* model that discriminates between NR and CR+PR in female patients. *GHRL* and *PEBP1* allowed the classification between responders (PR and CR) and NR in female patients. The details of the model subprocesses are presented in the table (A). The graph represents the distribution of the samples in a 2D plot. The blue line is the mathematical function defined by the values of the classifier (B). The Receiver Operating Characteristic (ROC) illustrates the performance of a our model as its discrimination threshold varies (C). Finally, the tables showing the performance in the sample separability tests allows for a clear evaluation of the model performing (D).
Abreviations: ACC (Accuracy), TP (True positives), TN (True negative), FP (False positive), FN (False Negative), PRE (Precision), SNS (Sensitivity), SPC (Specificity).

Supplementary Figure S7

A

Selected Strategy

Feature Selection Method	Relieff
Base classifier	Multilayer perceptron (MLP)
Ensemble	*Not applied*
Cost function	Balanced accuracy
Validation	10 K-fold

B

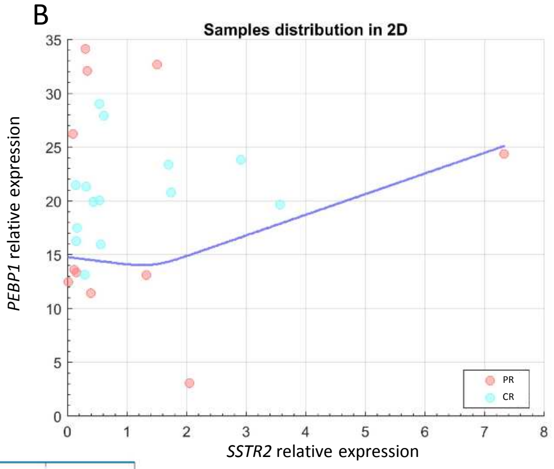

C

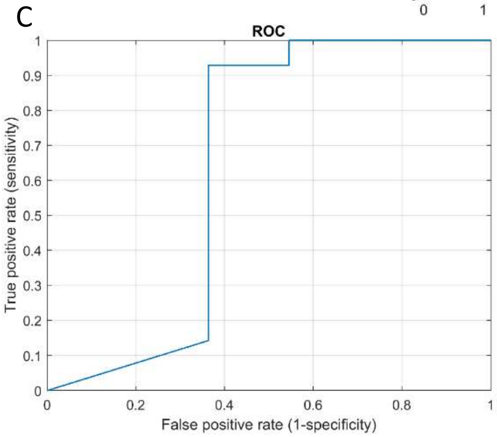

D

Samples separability test: Cross-validated

ACC	ACC p-value	TP	TN	FP	FN	PRE	SNS	SPC	Balanced ACC
76.00%	0.016	12	7	4	2	75.00	85.71	63.64	0.75

Samples separability test: without cross-validation

ACC	ACC p-value	TP	TN	FP	FN	PRE	SNS	SPC	Balanced ACC
80.00%	0.004	13	7	4	1	76.47	92.86	63.64	0.78

Supplementary Figure S7. ***SSTR2* + *PEBP1* model that discriminates between CR and PR in female patients.** *SSTR2* and *PEBP1* allowed the classification between CR and PR in female patients. The details of the model subprocesses are presented in the table (A). The graph represents the distribution of the samples in a 2D plot. The blue line is the mathematical function defined by the values of the classifier (B). The Receiver Operating Characteristic (ROC) illustrates the performance of a our model as its discrimination threshold varies (C). Finally, the tables showing the performance in the sample separability tests allows for a clear evaluation of the model performing (D).

Abreviations: ACC (Accuracy), TP (True positives), TN (True negative), FP (False positive), FN (False Negative), PRE (Precision), SNS (Sensitivity), SPC (Specificity).
